# Non-pharmacological pain management practice and barriers among nurses working in Debre Tabor Comprehensive Specialized Hospital, Ethiopia

**DOI:** 10.1371/journal.pone.0253086

**Published:** 2021-06-15

**Authors:** Shegaw Zeleke, Amare Kassaw, Yeshambaw Eshetie

**Affiliations:** 1 Department of Adult Health Nursing, College of Health Sciences, Debre Tabor University, Debre Tabor, Ethiopia; 2 Departments of Pediatrics and Child Health Nursing, College of Health Sciences, Debre Tabor University, Debre Tabor, Ethiopia; Prince Sattam Bin Abdulaziz University, College of Applied Medical Sciences, SAUDI ARABIA

## Abstract

**Background:**

Pain is an unpleasant sensory and emotional experience associated with or resembling that actual or potential tissue damage. Different study findings show that about 55% to 78.6% of inpatients experience moderate-to-severe pain. Nurses are one of the health professional who may hear of pain suffered by the patients and who can manage patient suffering by themselves. Therefore, their correct skill is very important in non- pharmacology and pharmacology pain management methods.

**Objective:**

To assess non-pharmacological pain management practice and barriers among nurses working in Debre Tabor Comprehensive Specialized Hospital, Ethiopia.

**Methods:**

Data were collected using structured observational check list with interviewer administered questionnaires that measure nurses’ practice on non-pharmacological pain management. Data were entered using Epi Data version 3.1 and analyzed using SPSS (Stastical Package for Social Sciences) version 23. Bivariable and multivariable analysis were conducted to examine the association between independent and outcome variables.

**Results:**

A total of 169 nurses participated in the study, with a response rate of 100%. Among the study participants 94 (55.6%) were females, and the mean age of nurses were 34.9(SD = 5.7) years. Only 44(26%) of nurses had good practice on non- pharmacology pain management methods. About 130(77.55%), 125(74.0%), and 123(72.8%) of nurses reported that inadequate cooperation of physicians, multiple responsibilities of nurses and insufficient number of nurses per patient ratio as barriers for practice of non -pharmacology pain management respectively.

**Conclusion:**

Majority of nurses didn’t apply non-pharmacological pain management practices for their patients in pain and the overall practice level of nurses was very poor. The major identified obstacle factors for the poor practice of non–pharmacological pain management methods were nurses’ fatigue, inadequate cooperation of physicians, heavy workload, multiple responsibilities of nurses, and insufficient number of nurses per patient ratio and unfavorable attitude of nurse on non-pharmacology pain management. Even if nurses experiences different challenges, they shall use non‐pharmacological pain management methods complementary to pharmacological treatment of pain as they are low cost and safe. And also boosting nurse’s attitude towards the effect of non–pharmacological pain management methods is crucial.

## Introduction

Pain is an unpleasant sensory and emotional experience associated with, or resembling that actual or potential tissue damage [1]. Pain also can be further defined as, whatever the patient says it is, and it exists whenever the patient says it does [2]. Studies have reported that 55% to 78.6% of inpatients experience moderate-to-severe pain. There are still problems regarding pain management despite countless training courses, application strategies, and multidisciplinary pain teams [3]. According to the American Nurses Association (ANA), the role of nurses in pain management includes the entire nursing process, assessment of pain, plan of pharmacologic and non-pharmacologic pain management strategies, implementation and evaluation of the response of the patient to the interventions [4]. Pain is the major symptom that brings patients to the healthcare setting and is also the commonest symptom with approximately 79% of hospitalized patients suffer from it [5,6]. Pain management practices are defined as a set of activities that should be provided by nurses to manage the patients’ pain effectively which includes assessing the patients’ pain, providing appropriate nurse’s interventions to relieve the patients’ pain and reassessing the patients’ pain after intervention [7,8]. According to the ANA, one of the role of nurses are managing patient pain based on non-pharmacologic pain management strategies such as psychological, educational and parental support [9,10]. Pain is influenced by psychological [11], cultural [12], social [13], and spiritual [14] factors. Even if non-pharmacological pain management do not replace pharmacological treatment, but these non-pharmacological pain management strategies used as a complementary to reduce symptoms, affect pain perception, assist with relaxation and to improve sleep pattern [15,16]. Specific non- pharmacological pain management strategies that Nurses can use to relief the suffering of patients in the health institution include music therapy, acupuncture, col/hot application, exercise, positioning therapy, massage therapy, social support, spiritual and religious support, hot and cold therapy, relaxation therapy, deep and slow breathing exercise and distraction therapy [15,17,18]. Nurses are one of the health professional who may hear of pain suffered by the patients and who can manage patient suffering by themselves. Therefore, their appropriate and accurate skill is very important in non- pharmacology and pharmacology pain management methods. Based on research studies in different areas of the country there is a knowledge and attitude gap on non-pharmacology pain management among practicing nurses in hospitals [19,20]. But, to the authors’ knowledge, it is hard to find studies that have been conducted in the study setting as well as in Ethiopia regarding to the practice of nurses on non-pharmacology pain management methods. So, these facts prompted the researchers to embark on the assessment of Nurses’ non-pharmacological pain management practice and barriers in Debre Tabor Comprehensive Specialized Hospital, Ethiopia.

## Methods

### Study setting and design

The study was carried out in Debre Tabor Comprehensive Specialized Hospital, which is found in North Central Ethiopia. Debre Tabor is located 50 kilometers east of Lake Tana and 665 kilometers from the capital city of Ethiopia, Addis Ababa. The study was conducted from October 1^st^ to December 30, 2020 with descriptive cross-sectional study design.

### Population and sample

The study populations were all nurses (N = 169) working in Debre Tabor Comprehensive Specialized Hospital wards(Medical ward, Surgical ward, Intensive Care Unit ward, Orthopedic ward, Pediatrics ward, Psychiatry ward, Emergency ward, Recovery ward). Since the study population was small, we did not determine the sample size rather census was conducted.

### Study variables, tools, and data collection procedure

The outcome variable was the practice of nurses on non-pharmacology pain management. The data were collected using observed structured cheek list with interviewer administered tools which contained socio-demographic questions, non-pharmacological pain management checklist and barriers that hindered the use of non-pharmacological pain management methods questions (S1 File). The tools were validated and adapted from a previous study with in-depth literature review [14,18,21,22]. The questionnaires were prepared in English and deployed for the respondents. The questionnaire was also checked thoroughly for objectivity and variable assessment before it was distributed to the data collectors. Half-day training was given to the data collectors and the supervisors on the study protocol, including the study objectives, the relevance of the study and confidentiality of information, respondent’s rights, and informed consent. One supervisor was a nurse holding Masters Science degree and two data collectors were nurses holding a Bachelor of Science degree. The scoring method for the outcome variable was adopted from a previous study [21]; good practice was recorded for nurses who apply non-pharmacology pain management methods routinely (≥75%).

### Ethics approval and consent to participate

An ethical clearance letter was obtained from a Research Ethics Committee of Debre Tabor University, College of health sciences (reference number: 972/2012 E.C./CHS). The permission and agreement consent was obtained from the study hospital prior to the study after a brief explanation of the purpose of the study through support letter. Informed written consent was obtained from all participants after a brief explanation of the aim of study. Confidentiality of information and privacy of participants’ was respected. The participants were told that information they provide use only for the purpose of this study. The names of the participants did not include questionnaire rather specific codes were used.

### Data processing and analysis

Data were entered into the computer using Epi Data version 3.1 and transported to Statistical Package for Social Sciences (SPSS) version 23.0 for analysis. Descriptive and inferential statistics were analyzed and presented. Initially, bivariate logistic regression was carried out to see the association of each independent variable with the outcome variable. Thereafter, to see the relationship of Nurses’ practice on non-pharmacology pain management, and socio-demographic and other variables multivariable logistic regression was used. Variables with a P-value ≤0.2 in the Bivariable logistic regression were used in the multiple logistic regression analysis. P-value ≤0.05 and 95% confidence level were considered as statistically significant.

## Results

### Socio-demographic characteristics of the nurses

A total of 169 nurses participated in the study, with a response rate of 100%. Among the study participants 94 (55.6%) were females, and the mean age of nurses were 34.9(SD = 5.7) years. About three fourth 125 (74%) of nurses marital status were married. Regarding educational status 132 (78.1%) were qualified with BSc degree in nursing, and only 24(14.2%) of nurses had training on non -pharmacology pain management **(Table 1).**

**Table 1:** Sociodemographic characteristics of nurses working in Debre Tabor Comprehensive Specialized Hospital, Ethiopia, 2020.

Variables	Frequency	Percent (%)
Age group in year		
≤25 years	4	2.4
26–35 years	102	60.4
36–45 years	56	33.1
46–55 years	7	4.1
Sex		
Male	75	44.4
Female	94	55.6
Marital status		
Single	34	20.1
Married	125	74.0
Divorced	7	4.1
Widowed	3	1.8
Educational level		
BSc nurse degree	132	78.1
Diploma nurse	37	21.9
Year of Nursing experiences		
≤5 years	45	26.6
5–10 years	73	43.2
10–20 years	35	20.7
≥20 years	16	9.5
Trained on non- pharmacology pain management		
Yes	24	14.2
No	145	85.8

### Nurses practice on non-pharmacological pain management methods

Regarding to nurses non pharmacology pain management practice 37(21.9%) of nurses apply movement restriction/resting, 31(18.3%) routinely use therapeutic communication with patient and family, 26(15.4%) apply hot or cold local packages routinely and 9(5.3%) of nurses provide quiet and comfortable room/reduce light intensity & alarms for the patients as non- pharmacology pain management. But, 169(100%) of nurses were never used acupuncture/acupressure as non-pharmacology patient pain management method. The overall non- pharmacology pain management practice level of nurses in Debre Tabor Comprehensive Specialized Hospital showed that only 44(26%) of nurses had good practice on non- pharmacology pain management methods (**Table 2**).

**Table 2:** Non-pharmacological pain management method utilization by nurses working in Debre Tabor Comprehensive Specialized Hospital, Ethiopia, 2020.

Variables	Frequency	Percent (%)
Repositioning		
Never	158	93.5
Routinely	11	6.5
Apply hot or cold local packages		
Never	159	84.6
Routinely	26	15.4
Apply breathing techniques		
Never	158	93.5
Routinely	11	6.5
Conduct Hydrotherapy/partial bathing		
Never	153	90.5
Routinely	16	9.5
Apply movement restriction/resting		
Never	132	78.1
Routinely	37	21.9
Therapeutic Communication with patient and family		
Never	138	81.7
Routinely	31	18.3
Use therapeutic touch		
Never	137	81.1
Routinely	32	18.9
Apply massaging techniques		
Never	143	84.6
Routinely	26	15.4
Distract the patient by listening light music/watching television		
Never	153	90.5
Routinely	16	9.5
Help the patient to pray		
Never	150	88.8
Routinely	19	11.2
Provide quiet & comfortable room/reduce light intensity & alarms		
Never	160	94.7
Routinely	9	5.3
Use comfort devices(special mattress)		
Never	159	94.1
Routinely	10	5.9
Counseling/provide education for patient and families		
Never	153	90.5
Routinely	16	9.5
Acupuncture/acupressure		
Never	169	100.0

### Barriers to the use of non-pharmacological pain management methods

Among the major obstacles or barriers identified by nurses for poor performance of non-pharmacological pain management for patients admitted in the ward; the following factors take the greatest responsibility. Based on nurses response around 121 (71.6%) said Nurses’ fatigue, 130(77.55%) inadequate cooperation of physicians, 121(71.6%) Nurses insufficient motivation to use non -pharmacology pain management methods because of low salary, about three fourth 125(74.0%) said heavy workload, 122(72.2%) disinclination and unwillingness to use non-pharmacological pain management, 125(74.0%) multiple responsibilities of nurses, 123(72.8%) an insufficient number of nurses per patient ratio and 113(66.9%) of nurse reported that inadequate training of nurses on non-pharmacological pain management methods (**Table 3).**

**Table 3:** Barriers to the use of non-pharmacological pain management methods among nurses working in Debre Tabor Comprehensive Specialized Hospital, Ethiopia, 2020.

Variables	Yes, N (%)	No, N (%)
Managers’ disinclination & noncooperation regarding the provision of non -pharmacology pain management	117(69.2)	52(30.8)
Inadequate facilities to use non-pharmacological methods	92(54.4)	77(45.6)
Infrequent use of non-pharmacological methods	115(68.0)	54(32.0)
Nurses’ fatigue	121(71.6)	48(28.4)
Inadequate nursing work experience and skills	110(65.1)	59(34.9)
Inadequate cooperation of physicians	123(72.8)	46(27.2)
Nurses insufficient motivation to use non -pharmacology pain management methods because of low salary	121(71.6)	48(28.4)
A heavy workload	125(74.0)	44(26.0)
Nurses insufficient knowledge about the latest research findings	118(69.8)	51(30.2)
Disinclination and unwillingness to use non-pharmacological	122(72.2)	47(27.8)
Nurses feeling that they are not adequately equipped to use such method	119(70.4)	50(29.6)
Multiple responsibilities	125(74.0)	44(26.0)
Nurses inadequate knowledge about the complications of inadequate pain management	118(69.8)	51(30.2)
Inaccessibility of proper pain assessment tools	111(65.7)	58(34.3)
An insufficient number of nurses in ward	123(72.8)	46(27.2)
Some patients’ inability to communicate and express pain	113(66.9)	56(33.1)
The high cost of some non-pharmacological methods	105(62.1)	64(37.9)
A chaotic environment	112(66.3)	57(33.7)
Inadequate training on non-pharmacological methods	113(66.9)	56(33.1)
Unstable health condition of patients	106(62.7)	63(37.3)
Nurses’ disinclination to use non-pharmacological methods	113(66.9)	56(33.1)
Insufficient knowledge about the pain behaviors of patients	107(63.3)	62(36.7)
Cultural differences between patients and nurses	103(60.9)	66(39.1)

Note: N = Number, % = percent.

### Factors associated with nurses’ practice on non-pharmacological pain management

In this study; Nurses who have non-pharmacology pain management training were three times (adjusted odds ratio [AOR] = 2.28, 95% CI = 1.14–6.32) more likely to have good practice on non-pharmacology pain management than nurses who didn’t have the training. Nurses who said that heavy workload and multiple responsibilities are an obstacle for the practice of non-pharmacology pain management were two times (adjusted odds ratio [AOR] = 2.61, 95% CI = 1.08–13.46) and three times (adjusted odds ratio [AOR] = 3.35, 95% CI = 1.42,-11.23) high likely to have good practice on non-pharmacology pain management than nurses who denies the obstacle respectively. Nurses with favorable attitude for non-pharmacology patient pain management were a positive predictor for good practice of non-pharmacology pain management (adjusted odds ratio [AOR] = 2.68, 95%CI = 2.13–8.01) (**Table 4).**

**Table 4:** Logistic regression analysis for practice of nurses’ on non-pharmacological pain management in Debre Tabor Comprehensive Specialized Hospital, Ethiopia, 2020.

Variables	Practice of nurses on non -pharmacology pain management		COR(95% CI)	AOR(95% CI)
	Poor, n (%)	Good, n (%)		
Sex				
Male	61(81.3)	14(18.7)	1	1
Female	64 (68.1)	30(31.9)	2.04(0.98, 4.23)	2.20(0.265, 18.25)
Educational level				
BSc nurse	96(72.7)	36(27.3)	1.34(0.56, 3.25)	4.60(0.53, 39.33)
Diploma nurse	29(78.4)	8(21.6	1	1
Trained on non -pharmacology pain management				
Yes	12(50)	12(50)	3.53(1.45, 8.61)*	2.82(1.14, 6.32)*
No	113(77.9)	32(22.1)	1	1
Inadequate cooperation of physicians				
Yes	83(67.5)	40(32.5)	5.06(0.69, 15.09)	4.26(0.34,12.19)
No	37(80.4)	9(19.6)	1	1
A heavy workload				
Yes	85(68.0)	40(32.0)	4.71(1.57, 14.05)*	2.61(1.08,13.46*
No	40(90.9)	4(9.1)	1	1
Multiple responsibilities				
Yes	97(75.8.0)	31(24.2)	4.70(1.58, 14.06)*	3.35(1.42, 11.23)*
No	36(87.8)	5(12.2)	1	1
An insufficient number of nurses in wards				
Yes	84(68.3)	39(31.7)	3.81(1.39, 10.38)*	2.86(1.17,9.24)*
No	41(89.1)	5(10.9)	1	1
Knowledge of nurses on non-pharmacology pain management				
Good knowledge	52(57.8)	38(42.2)	8.89(3.50, 22.56)*	7.27(2.96, 20.14)*
Poor knowledge	73(92.4)	6(7.6)	1	
Attitude of nurses on non-pharmacology pain management				
Favorable attitude	16(26.7)	44(73.3)	4.13(2.74, 9.15)*	2.68(2.13,8.01)*
Unfavorable attitude	94(94.5)	15(5.5)	1	

Note: n = number, % = percent, CI = confidence interval, COR = crude odds ratio, AOR- adjusted odds ratio.

## Discussion

In Ethiopia, few studies have assessed the practice of nurses’ regarding to non-pharmacological pain management; hence, the goal of this study was to assess the practice of nurses’ towards non-pharmacological pain management among nurses working in Debre Tabor Comprehensive Specialized hospital.

This study revealed that only 26% of nurses had good practice on non-pharmacological pain management. Our research result is similar with research done in Cairo University Hospital, Egypt the majority of nurses didn’t use non- pharmacological pain management methods/didn’t have good practice [23] and 40% of nurses in Illinois Wesleyan University have good practice on non-pharmacological pain management [24]. In our study setting for the poor implementation of non-pharmacological pain management methods; nurses’ infrequent training on non-pharmacological pain management methods, nurse’s unfavorable attitude towards non-pharmacological pain management, incorporation of physicians with nurses, poor nursing process implementation are the major accountable obstacles. The present finding shows that nurse’s experiences shortage of time due to increase work load and multiple responsibilities therefore nurses simply use pharmacological pain management protocol rather than using these therapeutic approaches of non-pharmacological pain management methods. This outcome is quite similar with study done at Makkah El-Mukarramah hospitals [25]. Regarding to nurses’ knowledge on non- pharmacological pain management methods 53.3% of participants had good knowledge. Our study finding is in congruent with research done at Benshangul Gumuz Hospitals 51.2% [20] and Iran Bindura Hospital 48.2% [26]. But, this study finding is lower compared with studies done in Zimbabwe 64.5% [27] and southern part of Ethiopia 78.1% [28]. The possible reasons may be variations in study setting, lack of training on non-pharmacological pain management, unwillingness of nurses to know the methods and disinclination of managers for non-pharmacological pain management. Regarding to nurses’ attitude this study showed that 35.5% of nurses’ have favorable attitude towards non-pharmacological pain management methods. It is lower than studies done at Benshangul Gumuz Hospitals 47% [20] and Iran Bindura Hospital 54% [26]. This discrepancy may be due to personal rating differences, sample size and socio demographic variation among the practicing nurses. In our study nurses with favorable attitude for non-pharmacology pain management methods were statistically significant associated with good practice of non-pharmacology pain management (adjusted odds ratio [AOR] = 2.68, 95%CI = 2.13–8.01). Moreover, nurses’ education showed that no statistically significant association with the practice of non-pharmacological pain management methods This finding is similar with research done in Egypt [21]. This similarity indicates that even the educational level of nurses has been increased, their practice on non-pharmacological pain management methods is low rather their attitude matters for their implementation. The major barriers that hindered non-pharmacological pain practices from being used by nurses were lack of time, nurses’ workload and multiple responsibilities of nurses. This result also congruent with the above research [21]. This similarity might be due to most African countries share similar characteristics in the health care system.

## Conclusion

Majority of nurses in Debre Tabor Comprehensive specialized Hospital didn’t apply non-pharmacological pain management practices for their patients in pain and the overall practice level of nurses were very poor. Nurses with favorable attitude had significant association with good practice of non–pharmacological patient pain management methods. So, boosting nurse’s attitude towards the effect of non–pharmacological patient pain management methods is crucial. The major identified obstacles factors for the poor practice of non–pharmacological patient pain management methods were nurses’ fatigue, inadequate cooperation of physicians, nurses insufficient motivation to use non -pharmacology pain management methods because of low salary, heavy workload, disinclination of the managers to use non-pharmacological pain management, multiple responsibilities of nurses, and an insufficient number of nurses per patient ratio. Therefore, theoretical and practical non-pharmacological pain management interventions should be incorporated in nursing curricula and advocating of the need and importance of non-pharmacological pain management methods. Moreover, regular dissemination of updated information to the nurses on these non-pharmacological pain management methods is recommended. And also they shall use non‐pharmacological pain management methods complementary to pharmacological treatment of pain as they are low cost and safe.

## Supporting information

S1 File Socio-demographic questions, non-pharmacological pain management checklist and barriers that hindered the use of non-pharmacological pain management methods questions. (DOCX)

## Abbreviations

ANA: American Nursing Association
BSc: Bachelor of Sciences
SPSS: Statistical Package for Social Sciences
