# Supplementary material for: Non-pharmacological pain management practice and barriers among nurses working in Debre Tabor Comprehensive Specialized Hospital, Ethiopia
Source: PLoS One. 2021 Jun 15;16(6):e0253086. doi: 10.1371/journal.pone.0253086 (PMC8205171; doi:10.1371/journal.pone.0253086)
Supplement: S1 File — (DOCX) [file pone.0253086.s001.docx]

**Questionnaires**

**Participant Information Sheet**

I am ___________. I would like to request you to participate in this research study. The aim of this study is to assess non-pharmacological pain management practice and barriers among Nurses working in Debre Tabor Comprehensive Specialized Hospital, Ethiopia. The purpose of this consent form is to give you information you will need to help you decide whether to participate in the study. The investigator will be available to answer any questions that arise during the study and afterwards. Any information that you provide will be held in strict confidentiality and will only be used only for the purpose of this study. No invasive procedures or tissue samples will be obtained from you as part of the study.

Participation in this study is purely voluntary and there is no monetary gain. You are free to withdraw from the study if you so wish without any penalty. No compensation will be offered for participation in the study.

**Consent form**

You are selected to be one of the participants in the study. The study will be conducted through interviewer administered questionnaire. We are asking you for a little of your time to help us in this study. In the end, it is hoped that the information you give us could help to design appropriate intervention. A code number will identify every participant and no name will be used. Your responses to any of the questions will not be given to anyone else and no reports of the study will ever identify you.

The interview is on voluntary basis. Your participation/ non-participation, or refusal to respond to the questions will have no effect now or in the future services or occupation that you have. Are you willing to participate in this study?

1. Yes. 2. No

If the answer is yes, thanks! Conduct the interview.

If the answer is no, Thanks! Proceed to the next eligible

Name and signature of interviewer ______________Date____________

Name and signature of supervisor ______________ Date_____________

**Part 1: Sociodemographic characteristics of Nurses**

| **S.No.** | **Variables** | **Response** | **Skip to** |
| --- | --- | --- | --- |
| 101. | Age | --------------years |  |
| 102. | Sex | 1. Male 2. Female |  |
| 103. | Marital status | 1. Single 2. Married 3. Divorced 4. Widowed |  |
| 104. | Educational level | 1. BSc nurse degree 2. Diploma nurse |  |
| 105. | Year of Nursing experiences | 1. ≤5 years 2. 5-10 years 3. 10-20 years 4. ≥20 years |  |
| 106. | Trained on non- pharmacology pain management | 1. Yes 2. No |  |

**Part II: Non-pharmacological pain management method utilization by Nurses**

| **S.No.** | **Variables** | **Response** | **Skip to** |
| --- | --- | --- | --- |
| 201. | Repositioning | 1. Never to sometimes 2. Often 3. Routinely |  |
| 202. | Apply hot or cold local packages | 1. Never to sometimes 2. Often 3. Routinely |  |
| 203. | Apply breathing techniques | 1. Never to sometimes 2. Often 3. Routinely |  |
| 204. | Conduct Hydrotherapy/partial bathing | 1. Never to sometimes 2. Often 3. Routinely |  |
| 205. | Apply movement restriction/resting | 1. Never to sometimes 2. Often 3. Routinely |  |
| 206. | Therapeutic Communication with patient and family | 1. Never to sometimes 2. Often 3. Routinely |  |
| 207. | Use therapeutic touch | 1. Never to sometimes 2. Often 3. Routinely |  |
| 208. | Apply massaging techniques | 1. Never to sometimes 2. Often 3. Routinely |  |
| 209. | Distract the patient by listening light music/watching television | 1. Never to sometimes 2. Often 3. Routinely |  |
| 210. | Help the patient to pray | 1. Never to sometimes 2. Often 3. Routinely |  |
| 211. | Provide quiet & comfortable room/reduce light intensity & alarms | 1. Never to sometimes 2. Often 3. Routinely |  |
| 212. | Use comfort devices(special mattress) | 1. Never to sometimes 2. Often 3. Routinely |  |
| 213. | Counseling/provide education for patient and families | 1. Never to sometimes 2. Often 3. Routinely |  |
| 213. | Acupuncture/acupressure | 1. Never to sometimes 2. Often 3. Routinely |  |

**Part III: Barriers to the use of non- pharmacological pain management methods**

| **S.No.** | **Variables** | **Response** | **Skip to** |
| --- | --- | --- | --- |
| 301. | Managers' disinclination & noncooperation regarding the provision of non -pharmacology pain management | 1. Yes 2. No |  |
| 302. | Inadequate facilities to use non-pharmacological methods | 1. Yes 2. No |  |
| 303. | Infrequent use of non-pharmacological methods | 1. Yes 2. No |  |
| 304. | Nurses' fatigue | 1. Yes 2. No |  |
| 305. | Inadequate nursing work experience and skills | 1. Yes 2. No |  |
| 306. | Inadequate cooperation of physicians | 1. Yes 2. No |  |
| 307. | Nurses insufficient motivation to use non -pharmacology pain management methods because of low salary | 1. Yes 2. No |  |
| 308. | A heavy workload | 1. Yes 2. No |  |
| 309. | Nurses insufficient knowledge about the latest research findings | 1. Yes 2. No |  |
| 310. | Disinclination and unwillingness to use non-pharmacological | 1. Yes 2. No |  |
| 311. | Nurses feeling that they are not adequately equipped to use such method | 1. Yes 2. No |  |
| 312. | Multiple responsibilities | 1. Ye 2. No |  |
| 313. | Nurses inadequate knowledge about the complications of inadequate pain management | 1. Yes 2. No |  |
| 314. | Inaccessibility of proper pain assessment tools | 1. Yes 2. No |  |
| 315. | An insufficient number of nurses in ward | 1. Yes 2. No |  |
| 316. | Some patients' inability to communicate and express pain | 1. Yes 2. No |  |
| 317. | The high cost of some non-pharmacological methods | 1. Yes 2. No |  |
| 318. | A chaotic environment | 1. Yes 2. No |  |
| 319. | Inadequate training on non-pharmacological methods | 1. Yes 2. No |  |
| 320. | Unstable health condition of patients | 1. Yes 2. No |  |
| 321. | Nurses' disinclination to use non-pharmacological methods | 1. Yes 2. No |  |
| 322. | Insufficient knowledge about the pain behaviors of patients | 1. Yes 2. No |  |
| 323. | Cultural differences between patients and nurses | 1. Yes 2. No |  |
